# The duality of self-harm and aggression: implications for research and practice

**DOI:** 10.1192/bjb.2025.10088

**Published:** 2025-08

**Authors:** Matina Shafti, Daniel Pratt, Peter Taylor, Andrew Forrester

**Affiliations:** 1 School of Human Sciences and Institute for Lifecourse Development, University of Greenwich, London, UK; 2 Division of Psychology and Mental Health, School of Health Sciences, Manchester Academic Health Science Centre, University of Manchester, Manchester, UK; 3 Division of Psychology and Mental Health, School of Health Sciences, Manchester Academic Health Science Centre, University of Manchester, Manchester, UK; 4 Division of Psychological Medicine and Clinical Neurosciences, School of Medicine, Cardiff University, Cardiff, UK

**Keywords:** Self-harm, aggression, violence, co-occurrence, dual harm

## Abstract

This editorial explores dual harm – the co-occurrence of self-harm and aggression – particularly among forensic populations. Historically approached as two separate and even opposing behaviours, emerging evidence shows that those who engage in self-harm and aggression experience greater adversity and poorer outcomes. This underscores the importance of enhancing our understanding of dual harm. We review key developments within the field, including how dual harm may be best conceptualised and managed, and identify critical gaps in the literature. In order to improve the care and outcomes of those who engage in self-harm and aggression, emphasis is placed on adopting more integrated approaches that consider the duality of these behaviours, as well as the complex needs of this high-risk group, within research and practice.

Self-harm and aggression have received extensive attention in the literature, with considerable research investigating their prevalence, characteristics and risk factors. However, one aspect remains relatively unexplored – their co-occurrence. Rather than engage in self-harm or aggression, some people engage in both – referred to as dual harm.^1^ Although the mere co-existence of behaviours does not necessarily warrant investigation, growing evidence highlights the importance of considering dual harm at empirical and clinical levels.

Self-harm and aggression have long been linked, with psychodynamic theorists considering self-harm as aggression turned inward. Yet, our understanding of their co-occurrence is limited. This is concerning, as individuals who engage in dual harm tend to experience worse outcomes than those who engage in self-harm alone or aggression alone (i.e. sole harm). For example, these individuals are more likely to experience premature death and use more severe methods of harm.^2,3^ Furthermore, dual harm is widespread among forensic populations, with a prevalence of up to 56% among forensic mental health patients, highlighting that this phenomenon is a pressing concern in forensic contexts.^4^

Adverse outcomes for individuals who engage in both self-harm and aggression are particularly evident in forensic settings. Although prisoners who dual harm represent a minority of the prison population, research suggests they spend longer in prison, face more disciplinary programmes and are responsible for nearly three-quarters of institutional misconduct incidents.^5^ They are also more likely to require treatment in medium or high/maximum-level security facilities.^6^ Such findings suggest that those who engage in both self-harm and aggression comprise a high-risk group, particularly in forensic contexts. Clinically, understanding dual harm is critical for identifying this group and developing effective interventions.

Prisoners, forensic mental health patients and institution staff have reported that self-harm and aggression among forensic groups are interrelated, sharing risk factors, functions and precipitating emotions.^7–10^ Qualitative research has found that prison staff viewed these behaviours as linked among prisoners, suggesting that, from staff’s perspective, both are rooted in the same emotions (e.g. anger, frustration).^7^ Despite the prevalence of co-occurring self-harm and aggression among forensic mental health patients and their suggested link, no established clinical guidelines exist for managing dual harm. This may lead to separate and incompatible approaches. Although self-harm is managed using a care-planning approach within forensic services, aggression tends to be addressed through restrictive practices, such as containment and punishment.^1^ However, the possible duality of self-harm and aggression, whereby both may arise from the same causal pathways and share functions, challenges the exclusive use of opposing strategies. Instead, integrated approaches that considers their interconnectedness (at a functional and manifest level) could lead to more effective care.

When investigating clinical phenomena, using an agreed construct definition is a valuable starting point to enable replicable, rigorous research. However, no agreed upon definition exists for dual harm. Researchers have primarily cross-tabulated separate measures of self-harm and aggression to assess dual harm, leading to methodological inconsistencies. Although some studies of dual harm assess a broad range of behaviours, others are more restrictive in their definitions. For example, in their study of dual harm, Steeg and colleagues^3^ assessed self-harm irrespective of suicidal intent, whereas Hemming and colleauges^11^ focused solely on suicidal behaviours. Moreover, it is unclear whether the proximity between harmful behaviours should factor into definitions.^12^ For instance, should the self-harm and aggressive behaviours occur within a short time, or could they co-occur at any point during an individual’s lifetime to be considered as dual harm? Such questions remain unanswered.

To our knowledge, Shafti and colleagues^13^ conducted the only study aiming to inform an evidence-based definition of dual harm. In the context of dual harm, predominant views perceive self-harm and aggression as linked.^14^ Therefore, we conducted a network analysis to assess which harmful behaviours are interrelated and should, therefore, be included in definitions of dual harm.^13^ The study assessed self-harm and various aggressive acts, including physical aggression, verbal aggression, property damage, arson, violence toward animals, dating violence and bullying. Findings revealed that relational aggression (bullying and dating violence) did not cluster with other harmful behaviours, suggesting these may be conceptually distinct.^13^ In line with findings that relational and non-relational forms of aggression have distinct correlates, it may be meaningful to exclude relational aggression in definitions of dual harm.^15^ However, it may be that self-harm and aggression are not linked in all instances of dual harm. Therefore, developing definitions based on this perspective alone may not reflect the true nature of this phenomenon.

Despite definitional challenges, the dual harm literature is steadily developing. Studies have primarily focused on comparing differences between those who engage in sole harm versus dual harm. Findings consistently show that the latter group is significantly more likely to present with various adverse social, psychological and environmental factors.^16–18^ For example, Richmond-Rakerd and colleagues^17^ found that compared with self-harm alone, dual harm was associated with greater childhood maltreatment, lower childhood self-control, difficulties with self-regulation, psychotic symptoms and substance dependence. Furthermore, compared with aggression alone, dual harm was significantly more likely to be associated with mental health problems and childhood victimisation.^18^ Such findings highlight that various risk factors exist at higher levels in those with a history of dual harm.

In line with the above evidence, it has been suggested that dual harm should be considered as a unique construct, comprising distinct risk factors from sole harm behaviours.^1^ This notion would be supported if certain factors are exclusively linked to dual harm, and not sole harm. However, a systematic review by Shafti and colleagues^12^ revealed insufficient evidence of psychological factors that are unique to dual harm. Instead, findings suggested that dual harm represents an overlap between self-harm and aggression, arising from the interaction and multiplicative effects of their risk factors. Similarly, Boxer^4^ hypothesised that dual harm arises from the accumulation (i.e. high loading) of personal and situational risk factors of self-harm and aggression. Supporting this, Carr and colleagues^16^ found that experiencing five or more childhood adversities was more prevalent in those with a history of dual harm (19.3%) compared with self-harm alone (10.9%) and aggression alone (11.4%). Moreover, as the number of adversities increased, so too did sole harm and dual harm, with the risk more than doubling for dual harm.^16^ Qualitative research has further revealed that prisoners and forensic mental health patients with a history of dual harm typically report various adversities throughout their lives, including lack of perceived social support, difficulties with emotional regulation and witnessing harmful behaviours.^9,10^ Such findings align with Boxer’s^4^ theory that those who dual harm have experienced a ‘high loading’ of risk factors.

Understanding the functionality of dual harm is crucial to understanding why individuals engage in self-harm and aggression. Qualitative research of prisoners and forensic mental health patients has suggested that this group struggle to manage negative emotions (i.e. emotional dysregulation), including identifying emotions and controlling their behaviours when distressed.^8–10^ As such, these individuals may engage in self-harm and aggression interchangeably to regulate their negative feelings (e.g. anger, sadness, frustration). This aligns with quantitative studies linking dual harm to emotional dysregulation across forensic and community-dwelling populations.^17,18^ In addition to emotional regulation, research has revealed that dual harm may further be used to communicate distress to others.^8,10^ Given evidence that co-occurring self-harm and aggression are linked to an accumulation of adverse events, and that these behaviours may be different manifestations of managing the resulting distress, trauma-informed care could be particularly important for those who dual harm.

The choice between self-harm and aggression at a specific point in time to fulfil such shared purposes may be influenced by the behaviour’s perceived usefulness (behavioural utility) and the individual’s ability to engage in it (behavioural accessibility).^9,10^ For example, in a study by Power and colleagues,^19^ prisoners reported choosing self-harm over violence because it achieved the same goal while avoiding the consequences of aggression within prison (e.g. punishment). Similarly, Gallagher and Sheldon^20^ found that 41% of forensic mental health patients reported self-harming as an alternative expression of aggression, suggesting that these behaviours serve the same purpose in these individuals. The choice of engaging in self-harm over aggression was primarily attributable to physical limitations (e.g. unable to harm others when in seclusion) and avoiding punishment. Therefore, identifying factors that affect the availability and utility of harmful behaviours (e.g. context, schemas) may inform management strategies of co-occurring self-harm and aggression. Doing so could also help identify individuals whose sole harm behaviours are at risk of developing into dual harm.

Although self-harm and aggression have been found to serve similar functions, they may also fulfil distinct purposes in those who engage in dual harm. The qualitative study by Shafti and colleagues^10^ revealed that although these behaviours had shared motivations among forensic mental health patients, at times, self-harm was also used as self-punishment because of feelings of worthlessness, and aggression as an act of defence, particularly within forensic settings where violence was perceived necessary for survival. Such findings highlight that although self-harm and aggression may be linked in the context of dual harm, this may not always be the case. Therefore, dual harm should be managed on a case-by-case basis, with particular attention given to the functions, links and divergence between the self-harm and aggressive behaviours.

Further research is needed to advance our understanding and management of dual harm within forensic and clinical settings. Establishing an agreed upon, evidence-based definition should be a priority within the literature. Moreover, including individuals with lived experience within research is crucial for developing meaningful and inclusive conceptualisations and interventions for dual harm. Rather than continuing predominant investigations that compare sole harm with dual harm, future investigations should explore the complex interplay and multiplicative effect of risk factors that may lead to dual harm, as well as the underlying functions of this phenomenon.

Individuals who dual harm often present with complex needs, requiring intensive support and placing significant demand on limited resources within forensic and clinical services. Interventions may be less effective without a more nuanced understanding of this high-risk group. Moving forward, a more integrated approach to assessment and intervention – one that acknowledges the duality of self-harm and aggression – could optimise resource allocation, improve risk management and enhance care. Greater recognition of dual harm should not only be a priority in research, but also a necessity within clinical practice to improve long-term outcomes for those who engage in self-harm and aggression.

## Data Availability

Data availability is not applicable to this article as no new data were created or analysed in its preparation.

## About the authors

**Matina Shafti** is a lecturer in forensic psychology at the University of Greenwich, London, UK, and an associate editor for the *International Journal of Forensic Mental Health*. **Daniel Pratt** is a professor of clinical psychology at the University of Manchester, Manchester, UK, and an Honorary Consultant Clinical Psychologist at Greater Manchester Mental Health NHS Foundation Trust, Manchester, UK. **Peter Taylor** currently works as a clinical reader at the University of Manchester, Manchester, UK. **Andrew Forrester** is Professor of Forensic Psychiatry at Cardiff University, Cardiff, UK, and Director of Research at Oxleas NHS Foundation Trust, Dartford, UK. He is Co-Chair of the Quality Network for Prison Mental Health Services and Editor-in-Chief of *BJPsych Bulletin*.
